# Using Feedback to Promote Physical Activity: The Role of the Feedback Sign

**DOI:** 10.2196/jmir.7012

**Published:** 2017-06-02

**Authors:** Jan-Niklas Kramer, Tobias Kowatsch

**Affiliations:** ^1^ Center for Digital Health Interventions Institute of Technology Management University of St. Gallen St.Gallen Switzerland

**Keywords:** feedback, internet, physical activity, health behavior, activity trackers

## Abstract

**Background:**

Providing feedback is a technique to promote health behavior that is emphasized by behavior change theories. However, these theories make contradicting predictions regarding the effect of the feedback sign—that is, whether the feedback signals success or failure. Thus, it is unclear whether positive or negative feedback leads to more favorable behavior change in a health behavior intervention.

**Objective:**

The aim of this study was to examine the effect of the feedback sign in a health behavior change intervention.

**Methods:**

Data from participants (N=1623) of a 6-month physical activity intervention was used. Participants received a feedback email at the beginning of each month. Feedback was either positive or negative depending on the participants’ physical activity in the previous month. In an exploratory analysis, change in monthly step count averages was used to evaluate the feedback effect.

**Results:**

The feedback sign did not predict the change in monthly step count averages over the course of the intervention (*b*=−84.28, *P*=.28). Descriptive differences between positive and negative feedback can be explained by regression to the mean.

**Conclusions:**

The feedback sign might not influence the effect of monthly feedback emails sent out to participants of a large-scale physical activity intervention. However, randomized studies are needed to further support this conclusion. Limitations as well as opportunities for future research are discussed.

## Introduction

### Background

In 2012, noncommunicable diseases (NCDs) such as diabetes, cardiovascular diseases, chronic respiratory diseases, or cancer were responsible for 68% of deaths worldwide [1].

Physical activity plays a crucial role in the prevention and management of NCDs, as it has been found to affect the incidence and course of NCDs such as diabetes [2], asthma [3], and cancer [4], as well as associated risk factors such as hypertension [5], overweight [6], or high blood sugar [7]. To reduce the burden of NCDs on the worlds’ health and health care systems, researchers have focused on the development of effective physical activity interventions among others.

Physical activity interventions often use feedback as a method to change behavior [8-11]. For example, in a review of technology-enabled health interventions [11], feedback was employed in 55 out of approximately 110 (50.0%) reviewed interventions targeting physical activity and was identified as the second-most used behavior change technique. Abraham and Michie [12] defined feedback as “providing data about recorded behavior or evaluating performance in relation to a set standard or others’ performance” (p.382). Despite its widespread use in physical activity interventions however, detailed analysis of feedback and its characteristics has so far been limited to behavioral domains other than health behavior, such as learning [13], professional care practice [14], or employee performance [15]. Research in these areas has indicated positive, yet highly variable effects of feedback on behavior [13-18]. For example, in their meta-analysis, Kluger and DeNisi [18] found a general positive effect of feedback on performance of mostly cognitive and motor tasks (*d*=.41), but in over one-third of all considered studies, feedback decreased performance. In a Cochrane review examining the effect of feedback on the compliance of health care professionals with desired practice, Jamtvedt et al [17] found a median increase in compliance of 5%. However, results varied from −16% to 70%. These results suggest the existence of further variables that may mediate or moderate the effect of feedback on behavior.

Behavioral theories provide a detailed specification of causal processes that lead to behavior change and can thus help to understand how feedback affects behavior [19]. Two different theories, control theory (CT) [20] and social-cognitive theory (SCT) [21], advocate the use of feedback, but both define different underlying processes. A better understanding of these processes can facilitate the design of feedback in physical activity (and other behavioral) interventions and can help to explain the variability of results of past research.

### Feedback According to Control Theory

**Figure 1:**
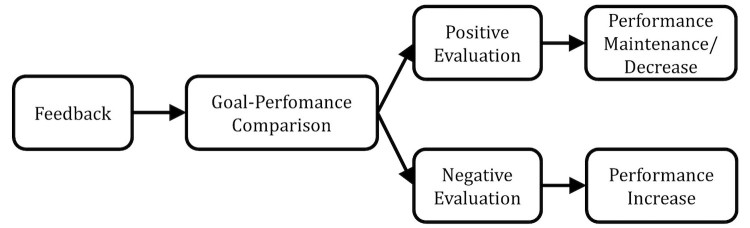
Effect of feedback according to control theory.

CT provides a model of self-regulation for intentional (or goal-directed) behavior (eg, walking 10,000 steps a day). Self-regulation is vital for physical activity promotion as it constitutes the basis for self-directed change [21], and physical activity interventions based on CT strategies have been found to be more effective than other physical activity interventions [22]. According to CT, people regulate their behavior by periodically comparing the perceived qualities of their own behavior with a salient reference value (eg, a goal) [23]. Whenever a discrepancy between one’s performance and a goal is recognized, a behavior is triggered in order to reduce the discrepancy (negative feedback loop). Thus, feedback affects behavior via the comparison with a set goal or standard, which has important implications for feedback design: performance is likely to be increased after negative feedback, as negative feedback informs the recipient that a goal or standard has not been met. In turn, performance is likely to be maintained or decreased after positive feedback, as positive feedback signals that the recipient exceeded the goal, which can thus be reached with less effort as well. Figure 1 illustrates the underlying causal processes of the effect of feedback according to CT.

### Feedback According to Social Cognitive Theory

In contrast to CT, SCT assumes that the mere perception of behavior and standards is insufficient to regulate behavior. It rather posits that cognitions such as self-efficacy beliefs are central factors that impact goal pursuit and self-regulation [24]. Self-efficacy beliefs are personal beliefs about the capability to exercise control over one’s actions and constitute the foundation of human motivation and action [24]. More precisely, unless one believes that a desired health outcome (eg, walking 10,000 steps a day) can be produced by one’s own actions, he or she has no incentive to act in the first place. Furthermore, self-efficacy beliefs influence the regulation of behaviors in a variety of ways. According to SCT, self-efficacy directly influences how goals are set and how strong one feels committed to achieving them, what outcomes one expects to realize, and how impediments are viewed [24]. It further affects how failures are attributed and how one reacts to setbacks [25]. People holding strong self-efficacy beliefs tend to be more motivated to reach set goals as they are convinced of their own capabilities, expect to realize favorable outcomes with their actions, and view impediments as surmountable.

**Figure 2:**
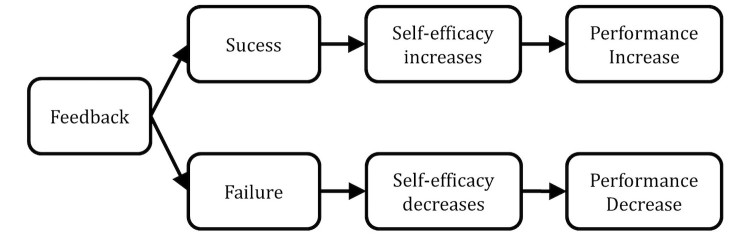
Effect of feedback according to social cognitive theory.

Understandably, a major source of self-efficacy includes personal experiences of success and failure [26]. Since feedback usually contains an evaluation of one’s performance with regard to a set goal, feedback conveys success if it is positive, that is, the recipient has achieved or exceeded the goal. In turn, feedback conveys failure if it is negative, that is, the recipient has not met the goal. Following the reasoning of SCT, performance is likely to increase after positive feedback because self-efficacy (and in turn performance) is increased. Consequently performance is decreased after negative feedback, which can undermine one’s confidence in the ability to reach a set standard. Figure 2 illustrates the underlying causal processes of the effect of feedback according to SCT.

### Research Question

Both CT and SCT contradict each other in their implications for feedback design. Whereas CT predicts that negative evaluation of performance leads to favorable behavior change, SCT predicts the same for positive evaluation of performance. Whether the feedback contains a positive or a negative evaluation of performance is often referred to as the sign of the feedback message [18]. Past research has so far produced inconclusive results regarding the effect of the feedback sign. In their meta-analysis, Kluger and DeNisi found no significant effect of the feedback sign, thus favoring neither of the two theoretical positions [18]. Both authors suggested later that the effect might be moderated by the regulatory focus of participants [27,28]. A more recent meta-analysis looking at the effect of feedback [29] could not evaluate the effect of the feedback sign, since too few studies directly compared positive and negative feedback. Consequently, it is unclear whether positive or negative feedback is more likely to change behavior when incorporated in physical activity interventions. Summarizing the line of reasoning above, we therefore pose the following research question: Does positive or negative feedback lead to more favorable behavior change in a physical activity intervention?

## Methods

### Procedure

In order examine the effects of positive and negative feedback, we exploratory analyzed data from a cluster-randomized trial that primarily focused on the effects of different incentives on the acceptance of a digital physical activity intervention [30]. This paper specifically examines the effect of monthly feedback emails that were sent out over the course of this intervention. The study was approved by the ethics committee of the authors’ university.

A total of 26,773 customers of a large Swiss health insurance company were invited through email, along with eligible family members, to participate in a physical activity intervention that was conducted from July 2015 to December 2015. In order to participate, customers had to be at least 18 years old, be registered in a complementary insurance program, accept participation conditions and privacy terms, and declare to be free of any medical condition that does not permit increased physical activity.

Before invitation, potential participants were clustered based on their state of residence and clusters were then randomly allocated to one out of three incentive conditions: In the financial incentive condition, participants received CHF10 (US $10) for each month they walked >10,000 steps a day on average. To prevent frustration, participants received CHF5 when their monthly step count average was below 10,000 but over 7500 steps, which matches the approximate minimum recommendation for daily physical activity [31]. The charitable incentive condition was equal to the financial incentive condition, except that participants could choose to donate an amount of choice of their earned money to a charitable organization. The control group received no incentives. To ensure fair treatment of all participants, the control group was entitled to financial incentives of CHF20 (for monthly averages above 10,000 steps) and CHF10 (for monthly averages below 10,000 and above 7500 steps) for the second half of the intervention. Participants completed a Web-based questionnaire at the beginning (T_1_) and at the end of the intervention (T_2_) to measure demographic and control variables relevant for the primary purpose of the study. A detailed description of the intervention as well as of all variables measured is available in Kowatsch et al [30].

In line with recommendations for health promotion, participants were advised to perform at least 150 min of moderate-intensity activity a week, which on average translates to a goal of 10,000 steps a day [31]. Via the Web-based customer account, participants could at any time gain insight into their physical activity data as well as their degree of achievement with regard to their goal of 10,000 steps a day.

**Figure 3:**
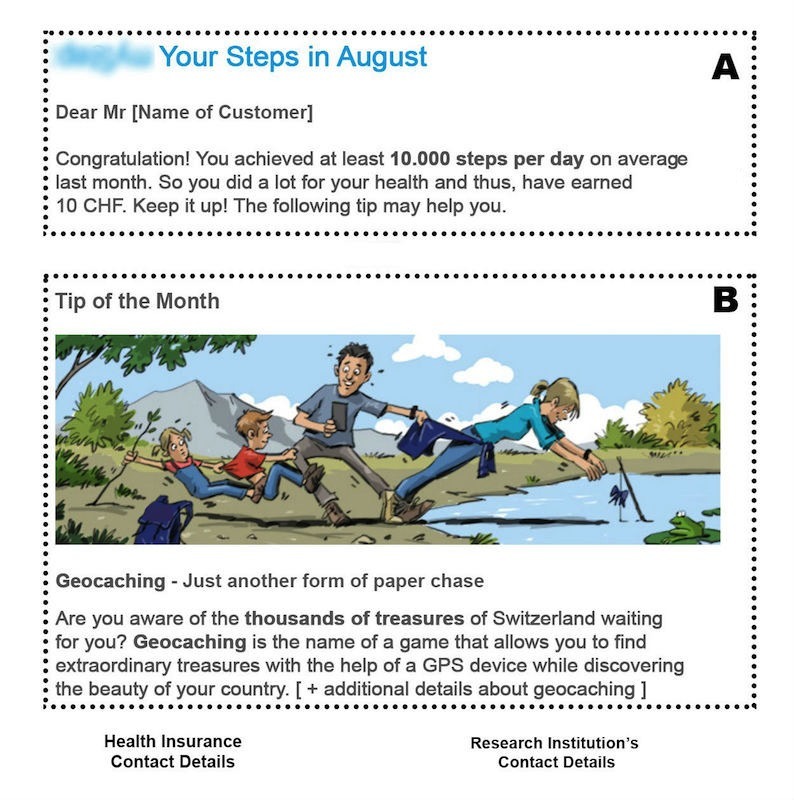
Exemplary feedback email (authors’ translation). A: Feedback message with positive or negative feedback depending on the performance of the participant. B: Season-based tip on how to increase physical activity (here: recommendation to participate in a geocaching activity).

### Feedback Emails

Starting after the first month, every participant received a feedback message by email at the beginning of the month that contained information on goal achievement of the last month. Consequently, every participant received 5 feedbacks over the course of the intervention. If participants failed to reach an average step count of at least 7500 steps a day, a negative feedback was provided (eg, “Unfortunately you did not reach the goal of 7500 steps a day on average last month”). In all other cases the feedback was positive (eg, “Well done, you have achieved at least 7500 steps a day on average over the last month and did a lot for your health”). In the financial and charitable incentive condition, feedback emails also contained information about the amount of money earned in the past month. Moreover, and in line with theory [18], all feedback emails contained a season-based tip on how to increase physical activity over the next month (eg, winter specific activities like snow shoe hiking or visiting Christmas markets were recommended in winter months, and outdoor activities like hiking or geocaching were recommended in summer and autumn months). Feedback emails were sent out by the insurance company. Figure 3 provides an example of a feedback email that was sent out in September.

### Analyses

#### Descriptive Statistics

Data from the baseline questionnaire was used to describe the sample of this study. We calculated means and standard deviations (SDs) for continuous variables and absolute and relative frequencies for categorical variables. Data on age and gender of the participants was provided by the insurance company. Statistics on monthly average step counts were obtained by calculating the mean of all participants’ mean step counts for each month.

#### Effect of the Feedback Sign

Because feedback on participants’ physical activity referred to monthly average step counts, we used the change in monthly step averages as the outcome variable to compare the effect of positive and negative feedback. Specifically, we calculated the difference between monthly step count averages before and after dispatch of the feedback email. Since each participant received 5 feedback emails, we consequently obtained 5 difference measures per participant. This difference indicates whether a participant increased or decreased his or her average monthly step count in the month after receiving feedback. Differences with an absolute value of more than 10,000 steps are likely to be the result of irregular recorded step counts (eg, very few and very low recorded step counts in 1 month) and were regarded as outliers and excluded from analysis. Exclusion of outliers resulted in the removal of 26/8115 (0.03%) observations and did not affect the results of the analyses.

To determine what analyses should best be used to examine the effect of the feedback sign, a 2-level hierarchical linear model with measurements as level-1 unit of analysis and participants as level-2 unit of analysis was fit to the data. However, the comparison of an intercept-only and a random-intercept model revealed that modeling the nested data structure did not significantly improve the model fit (χ^2^_1_=0.0, *P*=.99). Hence, measurements were treated as independent, and a linear regression model was fit to the data in order to examine the effect of the feedback sign. Because the feedback emails informed participants in the incentive groups also about the reception of a financial incentive, the main effect of the experimental group was included in the model. To further account for confounding effects of seasonality and regression to the mean, the model was adjusted for the effects of time and participants’ baseline physical activity [32]. In line with Barnett et al [32], baseline physical activity was defined as the average step count of the month preceding the dispatch of the feedback mail.

## Results

### Descriptive Statistics

In total, 1410 directly invited customers and 213 family members participated in the program resulting in a sample of N=1623 participants. On average, participants were 42.66 (SD=13.06) years old and slightly more men (848/1623, 52.25%) than women (770/1623, 47.44%) participated in the prevention program. Five participants (0.31%, 5/1623) did not disclose their gender. Compared with the Swiss population [33], people aged 20-39 years were overrepresented in the sample (46.40% [753/1623] vs 33.45% [2,225,129/6,651,623]), whereas people aged 64 years and above were underrepresented (7.64% [124/1623] vs 22.48% [1,495,052/6,651,623]). Moreover, program participants who filled out the Web-based questionnaire at T_1_ (n=1220) tend to have higher net income (between CHF 5000 and 10,000: 61.4% [593/966] vs 33.5%, more than CHF 10,000: 13.2% [127/966] vs 3.5%; no absolute values were available for the different income categories) and a lower chance of not obtaining a high school degree (2.30% [27/1172] vs 12.09% [564,889/4,671,164]; education data from the Federal Bureau of Statistics was available only for persons aged between 25 and 64 years and participants’ characteristics were adjusted accordingly). Additional participant characteristics obtained via the baseline questionnaire are outlined in Table 1.

Over the course of the intervention, participants walked on average 10,410 steps a day (Table 2), which can be considered high [31] but comparable with the results of other studies conducted with Swiss samples [34]. Monthly average step counts showed a slight negative trend over time. Naturally, step counts were higher for participants receiving a positive feedback mail because a positive feedback was sent out only if the average step count of the previous month exceeded 7500 steps. The attrition rate (23.35%, 379/1623) was comparable with previous pedometer-based interventions [35].

### Effect of the Feedback Sign

Descriptively, a clear pattern emerged from the data as it is apparent from Table 3. For every feedback mail, step count averages decreased after a positive feedback and increased after a negative feedback. In absolute terms, the average increase after negative feedback (450 steps, SD=2032) was comparable with the average decrease after positive feedback (−425 steps, SD=1858). For emails 2 and 3, the negative change after a positive feedback was less pronounced whereas for those emails the positive change after a negative feedback was almost twice the amount of steps when compared with emails 1, 4, and 5. However, mean changes in step counts were accompanied by large SDs, calling into question whether positive and negative feedback indeed led to significantly different changes in step counts. Moreover, the observed pattern of results can possibly be explained by a regression to the mean due to the dependency of the feedback sign on the participants’ physical activity.

**Table 1:** Participant characteristics.

Characteristics		T_1_ questionnaire, n (%) (n=1220)
**Education**		
	University	548 (44.92)
	Professional school	208 (17.05)
	High school	389 (31.89)
	Secondary school	23 (1.89)
	Primary school	4 (0.33)
	Not declared	48 (3.93)
**Place of residence**		
	Town	142 (11.64)
	Outskirts of town	300 (24.59)
	Village	598 (49.02)
	Countryside	180 (14.75)
**Income in CHF**		
	<2500	62 (5.08)
	2501-5000	184 (15.08)
	5001-7500	383 (31.39)
	7501-10,000	210 (17.21)
	>10,000	127 (10.41)
	Not declared	254 (20.82)
**Nationality**		
	Swiss	1098 (90.00)
	German	55 (4.51)
	Other	53 (4.34)
	Not declared	14 (1.15)
**Pedometer brand**		
	Fitbit	782 (64.10)
	Fitbit app	249 (20.41)
	Garmin	130 (10.66)
	Jawbone	59 (4.84)
**Pedometer bought for participation**		
	Yes	673 (55.16)
	No	511 (41.89)
	Not declared	36 (2.95)

**Table 2:** Descriptive statistics of monthly average step counts by feedback sign.

	Monthly step count average (SD)			Dropout, n (%)	
	Positive feedback^a^	Negative feedback^a^	Total^a^		
Month 1	-	-	10967.02 (3744.64)		53 (03.27)
Month 2	11581.79 (3273.48)	6293.99 (2668.99)	10710.19 (3732.68)		68 (04.19)
Month 3	11639.40 (3145.38)	6470.87 (2298.96)	10714.99 (3597.49)		65 (04.00)
Month 4	11533.57 (3326.82)	6450.72 (2269.40)	10657.20 (3717.10)		53 (03.27)
Month 5	11216.21 (3395.22)	6046.97 (1945.79)	10366.47 (3742.52)		91 (05.61)
Month 6	11308.43 (4283.81)	5968.97 (2144.36)	10299.51 (4479.57)		49 (03.02)
Total	11462.60 (3489.37)	6252.79 (2291.78)	10409.96 (3427.29)		379 (23.35)

^a^Values represent monthly average step counts depending on the feedback at the beginning of the month.

**Table 3:** Descriptive statistics of change in monthly step count averages by feedback sign.

	Mean difference in step counts (SD)		
	Positive feedback^a^	Negative feedback^a^	Total^a^
Mail 1	−482.71 (2035.95)	363.30 (2114.51)	−349.37 (2070.88)
Mail 2	−328.77 (1858.73)	717.76 (2153.88)	−148.82 (1952.29)
Mail 3	−399.11 (1869.87)	587.71 (2012.82)	−234.52 (1929.12)
Mail 4	−497.77 (1854.33)	215.26 (1654.28)	−382.55 (1842.09)
Mail 5	−422.96 (1607.90)	329.19 (2112.57)	−285.12 (1735.20)
Total	−425.34 (1858.38)	450.29 (2032.06)	−279.51 (1916.24)

^a^Values represent the mean change in monthly average step counts after dispatch of the feedback mail.

**Table 4:** Summary of multiple regression results predicting change in average step counts.

Model parameter	*b*	Standard error (*b*)	Beta	*P*
Intercept	−139.97	98.39	-	.16
Time	−24.98	15.84	−0.018	.12
Baseline physical activity^a^	−0.13	0.01	−0.239	<.001
Group: financial incentives^b^	3.71	80.30	.002	.96
Group: charitable incentives^b^	5.42	81.85	.001	.95
Feedback sign^c^	−84.28	78.74	−0.062	.28

^a^Baseline physical activity was centered before entering the model.

^b^Group membership was represented as 2 dummy variables with the control group serving as the reference group.

^c^Feedback sign was represented as 1 dummy variable with negative feedback serving as the reference group.

**Figure 4:**
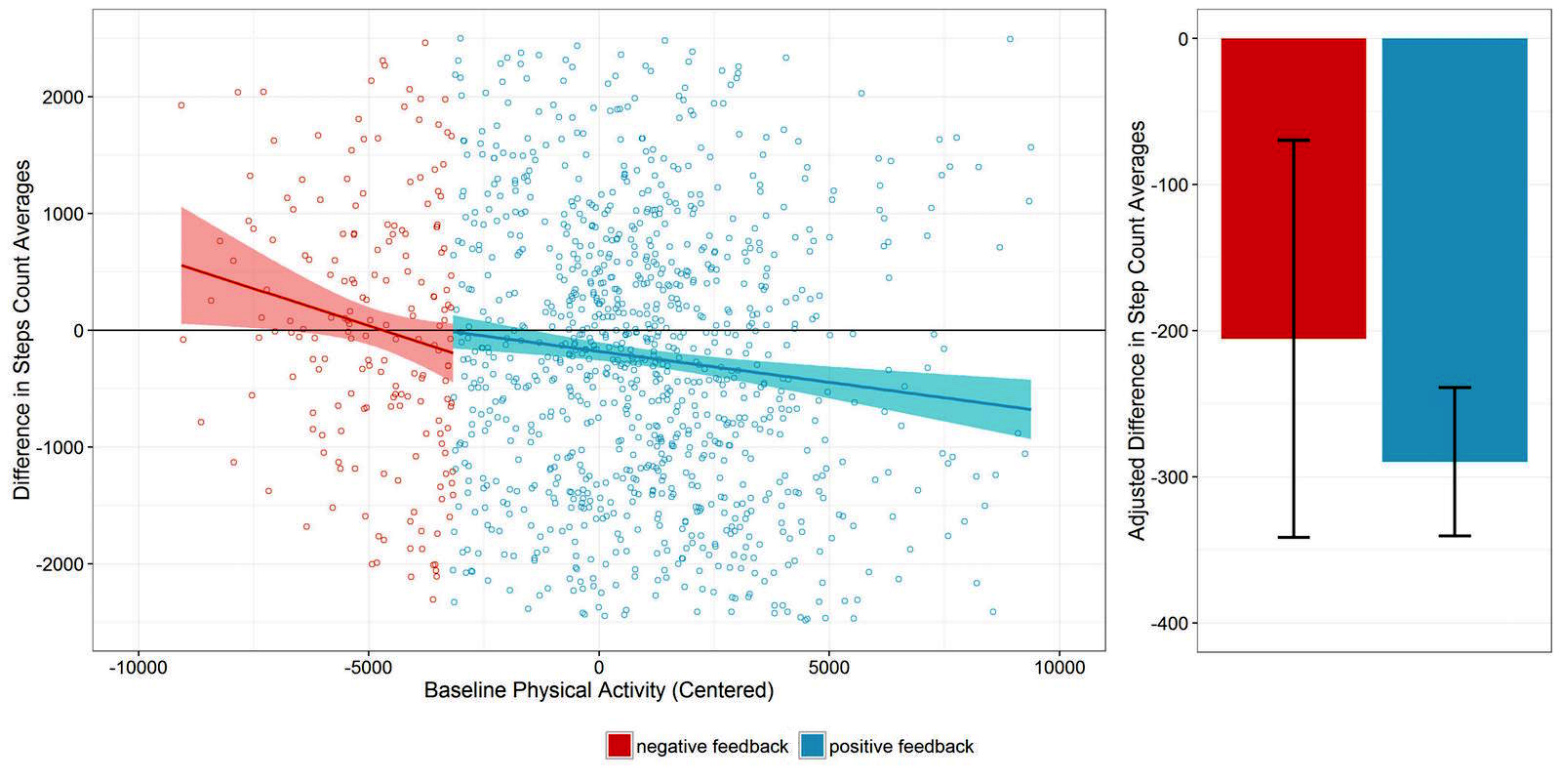
Left: Scatterplot of changes in monthly step counts against baseline physical activity for the first feedback email; the dark solid line represents perfect agreement (no change) and the colored lines are regression lines for positive and negative feedback. Right: Overall difference between negative and positive feedback emails adjusted for baseline physical activity and other covariates.

Results of the multiple regression analysis of change in monthly step counts on time, baseline physical activity, group, and feedback sign are presented in Table 4. Overall, the linear model significantly predicted the changes in monthly average step counts (*F*_5,6836_=90.84, *P*<.001) with *R*^2^=.062. Baseline physical activity emerged as the only significant predictor of change in step counts. The feedback sign did not significantly predict the change in monthly average step counts when controlling for the effects of time, group, and baseline physical activity (Figure 4, right plot), indicating that the descriptive patterns in Table 3 were caused by regression to the mean.

Figure 4 helps to further disentangle the effects of the feedback sign and baseline physical activity. The scatterplot in Figure 4 plots the dependent variable against participants’ baseline physical activity to illustrate the influence of regression to the mean using data from the first feedback email [32]. The colored lines represent separate regression lines for participants that received positive and negative feedback. The slopes of the regression lines indicate that the change in physical activity following the feedback mail is dependent on physical activity in the previous month for both positive and negative feedback. More specifically, changes in physical activity occurred predominantly for individuals with extreme baseline values. In line with the assumption of regression to the mean being the cause of the observed change, participants with very low baseline activity levels increased their activity in the following month, whereas participants with very high baseline levels showed a decrease in the following month. The descriptively observed difference between positive and negative feedback vanishes when controlling for baseline physical activity (Figure 4, right plot).

## Discussion

### Principal Findings

In this paper we analyzed the effect of positive and negative feedback emails on physical activity of participants of a large-scale physical activity intervention. Using a quasi-experimental approach, we found no difference between the effect of positive and negative feedback emails. Substantial differences found on a descriptive level could be explained by regression to the mean. Contrary to the theory outlined in this paper, our results might suggest that the feedback sign does not influence the effect of feedback. Similar results have been found by previous research in other behavioral domains [18]. However, due to the quasi-experimental setting and possible alternative explanations for our results, we refrain from drawing firm conclusions regarding our research question.

Both frequency and relevance of the feedback could have limited the general effect of feedback on participants’ physical activity levels in our study, thereby, explaining the missing effect of the feedback sign. Feedback in our study was only provided once at the beginning of each month, which might have not been frequent enough to affect monthly physical activity levels. Indeed, meta-analyses of feedback interventions in the area of health care demonstrate that feedback is more effective if it is delivered more frequently, for example, weekly [14]. Moreover, although the feedback messages contained novel and potentially interesting information (such as the final confirmation of earning a reward or tips on how to increase physical activity levels), the relevance of the feedback messages may have been compromised due to the permanent opportunity for participants to receive information on their step counts via their Web-based customer account or their activity tracker. Because irrelevant feedback is less likely to be processed by the recipient [36], this as well could have limited the effectiveness of the feedback emails. Including further personal information that is not yet provided by the activity tracker (eg, social comparisons) could enhance relevance of the feedback messages and might reveal differences between positive and negative feedback.

### Limitations

Some methodological issues arise as part of the practical setting of our study. Participants were not randomly allocated to a negative feedback and a positive feedback condition. Rather, positive and negative feedback was dependent on participants’ physical activity. As a consequence, we must not infer causality as inherent group differences beyond the included control variables may have affected our results. Furthermore, the internal validity of our results is limited as we were not able to check whether participants actually read the feedback emails. If a substantial proportion of participants ignored the feedback, we might be able to observe an effect of the feedback sign only within a subgroup of our sample that read the feedback emails. Finally, we were not able to include a true control group that did not receive any feedback emails. However, providing evidence for the general effectiveness of feedback was not the primary focus of this paper as this has been investigated and confirmed in other studies [8,14,18]. Feedback represents a widely used technique of physical activity interventions and is likely to be continuously used in the future due to the central role of self-regulation for both theoretical models of behavior change [20,21,37] and the practical effectiveness of physical activity interventions [22,38]. We therefore chose to focus on the comparison between positive and negative feedback to explore the way to best incorporate feedback into physical activity and health behavior change interventions.

### Future Research

Research regarding the effect of feedback in health behavior change interventions is in its infancy. Thus, we urgently call for the conduction of randomized controlled trials examining the effects of feedback on health behavior as well as related mediators and moderators. Results of those studies can help researchers and practitioners to decide how to best incorporate feedback in their health behavior interventions and thereby ensure a positive effect of feedback. In this context, digital technology might be a promising resource to maximize the effect of feedback [29]. Digital technology not only allows the delivery of automated and personalized feedback [39], sensors incorporated in mobile phones and wearables also facilitate so called just-in-time adaptive interventions (JITAIs) [40]. JITAIs are digital interventions that provide support to the user considering his or her state of vulnerability, opportunity, and receptivity. In other words, JITAIs provide support only to those persons who need it at the exact moment when they need it and can most benefit from it. Using the mobile phone, for example, feedback messages could be sent out only when the recipient is likely to actively process the feedback message (eg, when she is at home and no activities are scheduled in her calendar). Identifying the right conditions and context for feedback to be most effective holds great potential for health behavior change interventions.

### Conclusions

There is no difference between the effect of positive and negative feedback emails that were sent out on a monthly basis in a large-scale physical activity intervention. Framing of the feedback in terms of success and failure may not be crucial when the feedback is given infrequently and in situations when individuals are likely to be aware of their levels of behavior. However, feedback characteristics, including the feedback sign, should be carefully considered when designing feedback to change health behaviors.

## Abbreviations

CT: control theory
JITAI: Just-in-Time adaptive Intervention
NCD: Non-communicable Disease
SCT: social-cognitive theory
SD: standard deviation

## References

[1] Mendis S, Armstrong T, Bettcher D, Branca F, Lauer J, Mace C, Poznyak V, Silva V, Stevens G (2014). Global status report on noncommunicable diseases.

[2] Pan XR, Li GW, Hu YH, Wang JX, Yang WY, An ZX, Hu ZX, Lin J, Xiao JZ, Cao HB, Liu PA, Jiang XG, Jiang YY, Wang JP, Zheng H, Zhang H, Bennett PH, Howard BV (1997). Effects of diet and exercise in preventing NIDDM in people with impaired glucose tolerance. The Da Qing IGT and diabetes study. Diabetes Care.

[3] Carson KV, Chandratilleke MG, Picot J, Brinn MP, Esterman AJ, Smith BJ (2013). Physical training for asthma. Cochrane Database Syst Rev.

[4] Ibrahim EM, Al-Homaidh A (2011). Physical activity and survival after breast cancer diagnosis: meta-analysis of published studies. Med Oncol.

[5] Siebenhofer A, Jeitler K, Horvath K, Berghold A, Posch N, Meschik J, Semlitsch T (2016). Long-term effects of weight-reducing drugs in people with hypertension. Cochrane Database Syst Rev.

[6] Shaw K, Gennat H, O'Rourke P, Del MC (2006). Exercise for overweight or obesity. Cochrane Database Syst Rev.

[7] Norris SL, Engelgau MM, Narayan KM (2001). Effectiveness of self-management training in type 2 diabetes: a systematic review of randomized controlled trials. Diabetes Care.

[8] Gardner B, Smith L, Lorencatto F, Hamer M, Biddle SJ (2016). How to reduce sitting time? a review of behaviour change strategies used in sedentary behaviour reduction interventions among adults. Health Psychol Rev.

[9] O'Brien N, McDonald S, Araújo-Soares V, Lara J, Errington L, Godfrey A, Meyer TD, Rochester L, Mathers JC, White M, Sniehotta FF (2015). The features of interventions associated with long-term effectiveness of physical activity interventions in adults aged 55-70 years: a systematic review and meta-analysis. Health Psychol Rev.

[10] Olander EK, Fletcher H, Williams S, Atkinson L, Turner A, French DP (2013). What are the most effective techniques in changing obese individuals' physical activity self-efficacy and behaviour: a systematic review and meta-analysis. Int J Behav Nutr Phys Act.

[11] Winter SJ, Sheats JL, King AC (2016). The use of behavior change techniques and theory in technologies for cardiovascular disease prevention and treatment in adults: a comprehensive review. Prog Cardiovasc Dis.

[12] Abraham C, Michie S (2008). A taxonomy of behavior change techniques used in interventions. Health Psychol.

[13] Hattie J, Timperley H (2007). The power of feedback. Rev Educ Res.

[14] Ivers NM, Grimshaw JM, Jamtvedt G, Flottorp S, O'Brien MA, French SD, Young J, Odgaard-Jensen J (2014). Growing literature, stagnant science? systematic review, meta-regression and cumulative analysis of audit and feedback interventions in health care. J Gen Intern Med.

[15] Alvero AM, Bucklin BR, Austin J (2001). An objective review of the effectiveness and essential characteristics of performance feedback in organizational settings (1985-1998). J Organ Behav Manage.

[16] Ivers N, Jamtvedt G, Flottorp S, Young JM, Odgaard-Jensen J, French SD, O'Brien MA, Johansen M, Grimshaw J, Oxman AD (2012). Audit and feedback: effects on professional practice and healthcare outcomes. Cochrane Database Syst Rev.

[17] Jamtvedt G, Young JM, Kristoffersen DT, O'Brien MA, Oxman AD (2006). Audit and feedback: effects on professional practice and health care outcomes. Cochrane Database Syst Rev.

[18] Kluger AN, DeNisi A (1996). The effects of feedback interventions on performance: a historical review, a meta-analysis, and a preliminary feedback intervention theory. Psychol Bull.

[19] Michie S, Abraham C (2004). Interventions to change health behaviours: evidence-based or evidence-inspired?. Psychol Health.

[20] Carver CS, Scheier MF (1982). Control theory: a useful conceptual framework for personality-social, clinical, and health psychology. Psychol Bull.

[21] Bandura A (1998). Health promotion from the perspective of social cognitive theory. Psychol Health.

[22] Michie S, Abraham C, Whittington C, McAteer J, Gupta S (2009). Effective techniques in healthy eating and physical activity interventions: a meta-regression. Health Psychol.

[23] Carver CS, Scheier MF (1990). Origins and functions of positive and negative affect: a control-process view. Psychol Rev.

[24] Bandura A (2004). Health promotion by social cognitive means. Health Educ Behav.

[25] Bandura A (1991). Social cognitive theory of self-regulation. Organ Behav Hum Decis Process.

[26] Bandura A (1977). Self-efficacy: toward a unifying theory of behavioral change. Psychol Rev.

[27] Van Dijk D, Kluger AN (2010). Task type as a moderator of positive/negative feedback effects on motivation and performance: a regulatory focus perspective. J Organiz Behav.

[28] Van-Dijk D, Kluger AN (2004). Feedback sign effect on motivation: is it moderated by regulatory focus?. Appl Psychol.

[29] Hermsen S, Frost J, Renes RJ, Kerkhof P (2016). Using feedback through digital technology to disrupt and change habitual behavior: a critical review of current literature. Comput Human Behav.

[30] Kowatsch T, Kramer J, Kehr F, Wahle F, Elser N, Fleisch E (2016). Effects of charitable versus monetary incentives on the acceptance of and adherence to a pedometer-based health intervention: study protocol and baseline characteristics of a cluster-randomized controlled trial. JMIR Res Protoc.

[31] Tudor-Locke C, Craig CL, Brown WJ, Clemes SA, De CK, Giles-Corti B, Hatano Y, Inoue S, Matsudo SM, Mutrie N, Oppert J, Rowe DA, Schmidt MD, Schofield GM, Spence JC, Teixeira PJ, Tully MA, Blair SN (2011). How many steps/day are enough? for adults. Int J Behav Nutr Phys Act.

[32] Barnett AG, van der Pols JC, Dobson AJ (2005). Regression to the mean: what it is and how to deal with it. Int J Epidemiol.

[33] (2017). Bundesamt für Statistik.

[34] Sequeira MM, Rickenbach M, Wietlisbach V, Tullen B, Schutz Y (1995). Physical activity assessment using a pedometer and its comparison with a questionnaire in a large population survey. Am J Epidemiol.

[35] Bravata DM, Smith-Spangler C, Sundaram V, Gienger AL, Lin N, Lewis R, Stave CD, Olkin I, Sirard JR (2007). Using pedometers to increase physical activity and improve health: a systematic review. JAMA.

[36] Petty R, Cacioppo J (1986). The elaboration likelihood model of persuasion. Adv Exp Soc Psychol.

[37] Schwarzer R, Luszczynska A (2008). How to overcome health-compromising behaviors. Eur Psychol.

[38] Conn VS, Hafdahl AR, Brown SA, Brown LM (2008). Meta-analysis of patient education interventions to increase physical activity among chronically ill adults. Patient Educ Couns.

[39] Yardley L (2011). The potential of Internet-delivered behaviour change interventions. The European Health Psychologist.

[40] Nahum-Shani I, Hekler EB, Spruijt-Metz D (2015). Building health behavior models to guide the development of just-in-time adaptive interventions: a pragmatic framework. Health Psychol.

